# Statistical learning of distractor locations is dependent on task context

**DOI:** 10.1038/s41598-023-38261-z

**Published:** 2023-07-11

**Authors:** Jasper de Waard, Dirk van Moorselaar, Louisa Bogaerts, Jan Theeuwes

**Affiliations:** 1 Department of Experimental and Applied Psychology, Vrije Universiteit Amsterdam, Van der Boechorststraat 7, 1081 BT Amsterdam, The Netherlands; 2 Institute Brain and Behavior Amsterdam (iBBA), Amsterdam, The Netherlands; 3 Department of Experimental Psychology, Ghent University, Ghent, Belgium; 4 William James Center for Research, ISPA-Instituto Universitario, Lisbon, Portugal

**Keywords:** Psychology, Human behaviour

## Abstract

Through statistical learning, humans can learn to suppress visual areas that often contain distractors. Recent findings suggest that this form of learned suppression is insensitive to context, putting into question its real-life relevance. The current study presents a different picture: we show context-dependent learning of distractor-based regularities. Unlike previous studies which typically used background cues to differentiate contexts, the current study manipulated task context. Specifically, the task alternated from block to block between a compound search and a detection task. In both tasks, participants searched for a unique shape, while ignoring a uniquely colored distractor item. Crucially, a different high-probability distractor location was assigned to each task context in the training blocks, and all distractor locations were made equiprobable in the testing blocks. In a control experiment, participants only performed a compound search task such that the contexts were made indistinguishable, but the high-probability locations changed in exactly the same way as in the main experiment. We analyzed response times for different distractor locations and show that participants can learn to suppress a location in a context-dependent way, but suppression from previous task contexts lingers unless a new high-probability location is introduced.

## Introduction

The traditional division of attentional selection into *top-down* (voluntary, goal-driven) and *bottom-up* (automatic, stimulus-driven) effects^1–4^ is making way for a third category, called *selection history*^5,6^. Selection history, which refers to learned attentional effects that neither can be explained by top-down nor by bottom-up processes, is studied predominantly in paradigms such as contextual cueing^7,8^, reward learning^9,10^, and as of recently statistical learning of target enhancement^11,12^ and distractor suppression ^11,13,14^. Though the full breadth of history-based effects is as of yet likely unknown, the presumed common denominator across all those effects is that past experiences of attentional selection are (often implicitly) learned, and put to use “when the relevant context is encountered”. The current work investigates the latter statement on context-dependency in relation to statistical learning of distractor suppression.

Statistical learning concerns the extraction of regularities in space and time from sensory input^15^. As a topic of research, it has gained a lot of momentum after the seminal discovery that infants can learn the transitional probabilities from one syllable to the next, facilitating word segmentation^16^. Since then, the focus has been extended to adults^15^, and the statistical learning paradigm was ported to the visual domain by replacing syllables with shapes^17,18^. Similarly, spatial relations between shapes and distributional regularities regarding shapes’ frequencies are readily picked up even during passive viewing^17,19,20^. In contextual cueing, statistical learning is observed as facilitated target detection when searching through earlier encountered display configurations compared to searching through novel displays^7,8,21^. Furthermore, in a probability cueing paradigm, target search is implicitly biased towards the location where the target appears most often; participants are faster when the target appears in the high-probability location compared to low-probability locations^22,23^. Particularly relevant for the present study is the learning of regularities regarding distracting stimuli. Learning the likely location of a distractor can help to decrease distraction, thereby facilitating target detection. Adapting the classic additional singleton paradigm^24,25^, Wang and Theeuwes^14^ introduced a statistical regularity in the location of the uniquely colored distractor, such that it was far more likely to appear in one location (the high-probability location) than any of the seven other (low-probability) locations in the search display. As a result, participants learned to suppress the high-probability location, as reflected by faster search times when the distractor appeared on the high-probability location and slower search times when the target appeared there^11,14,26–29^.

Context plays a major role in many theories of learning and memory^30^, and for good reason as the advantages of context sensitivity are large. Without some form of context sensitive learning, selection history effects would require a constant re-learning of biases for contexts that have been encountered before, and perhaps more catastrophically, inadequate biases would persist even when they are no longer relevant. Indeed, many of the empirical findings align with the advantages of context dependency. In visual search, contextual cueing and reward learning have been shown to involve context-dependent learning^31–35^. For example, stimulus features that have been rewarded in a particular context, later only captured attention when presented in the same context (e.g., same background scene^31^). In contextual cueing, *hyper specificity* was reported, with no transfer at all between contexts that only differed in color^35^. Furthermore, if background images are associated with search displays that require either feature search (searching for a specific combination of features) or singleton search (searching for a deviation from the rest) in a training phase, those backgrounds reinstate the associated search strategy in a testing phase^36,37^. Context-dependent distractor effects have also been observed in habituation-based studies^38,39^. After repeated exposure, attentional capture for the distractor habituated (decreased), but returned in full a day later when the background was changed, while it remained habituated when the background was kept the same^39^. However, habituation in these experiments was not location-specific.

Beyond visual search, context-dependent effects have also been found in sequence learning studies. For example, in an fMRI study on explicit sequence learning of object images, two contexts (cued by the color of the fixation dot) were associated with different sequences while using the same objects (i.e. ABC versus ACB)^40^. The results showed progressively faster responses to context-matching versus mismatching sequences, as well as context-specific expectation suppression in the BOLD response. Furthermore, contextual cues such as a change in voice or pitch help listeners to track multiple sets of embedded patterns in continuous speech input^41,42^. Without such a cue to signify a change in context, previously learned patterns are rapidly replaced by new ones^43^.

Context-dependent distractor suppression would make it possible to revive suppression of potentially distracting stimuli when a given context is encountered again, and to extinguish previous suppression settings when the context changes. In other words, previously learned suppression could be applied selectively to match a particular context. By contrast, context-independent distractor suppression would require constant learning and unlearning of suppression to adapt to the current situation, necessarily lagging behind because sufficient repetitions are required to update the suppression settings. Taken together, the theoretical benefits of context-dependency as well as the observed context-dependent results in related paradigms provide a case for context-dependent distractor suppression.

Counter to this idea however, using an adapted version of the distractor-based statistical learning paradigm of Wang and Theeuwes^14^, Britton and Anderson^44^ reported suppression effects that were not context specific but instead generalized across contexts. In their study (Experiment 1), the context on each trial was determined by a grayscale background image of a forest or a city (as in a prior study by Anderson on reward learning^31^). Crucially, the high-probability distractor location depended on the context, so that the urban background predicted a different distractor location than the forest. Furthermore, the context was visible well before the onset of the search display, so that the most probable upcoming distractor location could in principle be anticipated. The results indicated that learning had taken place: search times were faster when the distractor was at a high-probability versus a low-probability location. However, between the two high-probability distractor locations, search times were the same irrespective of the context. Motivated by the discrepancy between theoretical predictions of context-dependency and empirical evidence for the absence thereof, de Waard et al.^45^ conceptually replicated these results across three experiments. Experiment 1 signified context through the color of the background. To increase the distinction between the two contexts, Experiment 2 employed an auditory versus a visual cue, motivated by findings from the temporal preparation literature^46^. In Experiment 3, each context was coupled with a different response mapping, so that processing the context was a necessity for performing the task. Throughout all these experiments, including Britton and Anderson’s^44^, Bayesian analyses indicated an absence of context-dependent suppression (only a group of participants that showed awareness of the regularities in Experiment 3 yielded some context-dependent effects). In other words, the suppression was not flexibly adjusted from context to context, but both high-probability distractor locations were equally suppressed regardless of the context.

Although these results appear to demonstrate context insensitivity during learning, one should consider that the different contexts were randomly intermixed. While context-dependent findings in reward or punishment learning^31,32,34^ were also obtained with intermixed designs, statistical learning necessarily requires repetitions across multiple trials, and it is possible that intermixed designs simply do not provide the required repetitions per context to develop context associations, such that different contexts end up lumped together. Conversely, reward and punishment learning may simply require fewer trials for learning to take place. Furthermore, a stronger context manipulation could uncover context-dependent effects that would remain hidden using only background manipulations. Therefore, we developed a paradigm in which the context was determined by the task. Half of the trials employed a compound search task, where participants responded to the orientation of the line inside the target. The other half employed a detection task, where participants responded to the absence or presence of the target, which has been shown to yield a pattern of response times that is very similar to the compound search task in its sensitivity to distractor regularities^47^. Crucially, a different high-probability distractor location was assigned to each task, and these two distractor locations were located opposite of each other^48^.

**Figure 1:**
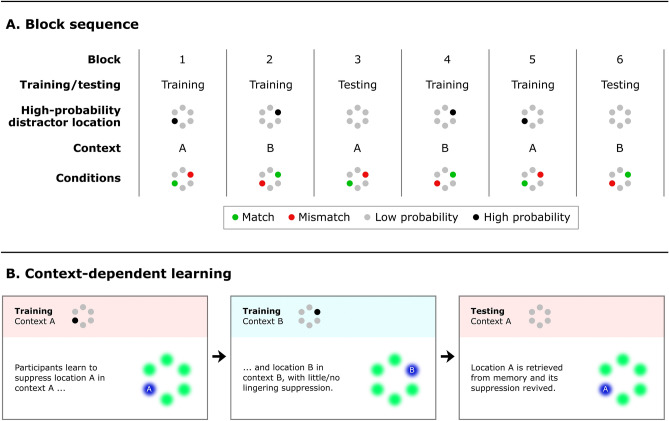
Illustration of the context and probability changes across blocks (**A**), as well as a graphical prediction of the results in the case of context-dependent learning (**B**). Figure created in Adobe Photoshop 22.4.3, https://www.adobe.com/products/photoshop.html.

The task context, denoted Context A and Context B, was blocked to allow enough time for context-location associations to be formed and later retrieved. A context-location association was formed in the first training block for context A and the second for context B. In the subsequent testing block, context A was reintroduced, but all distractor locations were made equiprobable, such that any space-based suppression can only be attributed to learning of the distractor location regularities during training. This three-block procedure was then repeated for another three blocks with opposite contexts, such that each context was tested in one block. The total block sequence is thus: training A, training B, *testing A*, training B, training A, *testing B* (see also Fig. 1A). If learning is indeed independent of experimental context (as previous studies suggest), the testing blocks should show lingering suppression that is most pronounced for the last-learned location. That is, in the first testing block one should observe lingering suppression at the location that had a high distractor probability in training context B, and in the second testing block at the high probability distractor location associated with context A. By contrast, if learning is context-dependent, there should be little to no lingering suppression, but instead a revived suppression from the associated context (Fig. 1B). To further characterize the context effect, we also ran a control experiment in which the spatial distractor balance shifted across blocks in the same way as the main experiment, but critically there were no longer different task contexts that could be linked to this distractor manipulation (i.e., the task stayed the same throughout the entire experiment).

## Main experiment

Statistically induced distractor suppression has been shown to occur within comparable time spans and experimental setups in compound search tasks, where participants respond to the line orientation inside the uniquely shaped target^14^, as well as visual detection tasks, where participants respond to the presence or absence of a uniquely shaped target^47^. In Experiment 1 we combined these variants of the additional singleton paradigm into a single experiment. We reasoned that they were different enough to induce a different task context, yet similar enough to be analyzed in a comparable way.

### Methods

#### Participants

Following Britton and Anderson^44^, an effect with d = 0.6 would require a sample size of 31 to get β = 0.90 when α = 0.05. However, since they did not find a significant result, we attempted to detect a smaller effect size (d = 0.45), which required a sample size of 54 to get β = 0.90 when α = 0.05. The number of non-discarded participants equaled or exceeded this minimal sample size in both experiments. Fifty-four adults (26 male, 27 female, 1 non-binary, mean age = 31, age range: 22 to 50) participated in Experiment 1 through Prolific^49^. They all reported having normal or corrected-to-normal (color) vision, and at minimum an undergraduate degree. Participation took approximately 35 min and participants earned £4,70. The experiment was approved by the Ethical Committee of the faculty of Behavioral and Movement Sciences of the Vrije Universiteit Amsterdam. Before the experiment, all participants gave informed consent and all the methods were performed in accordance with the Declaration of Helsinki.

#### Apparatus and stimuli

Because the experiment took place online, some factors (e.g. lighting and seating conditions) could not be controlled. For replication purposes, item sizes and colors are reported in pixels and RGB values (red/green/blue). The experiment was created in OpenSesame^50^ using OSweb 1.4.11, and run using JATOS^51^.

**Figure 2:**
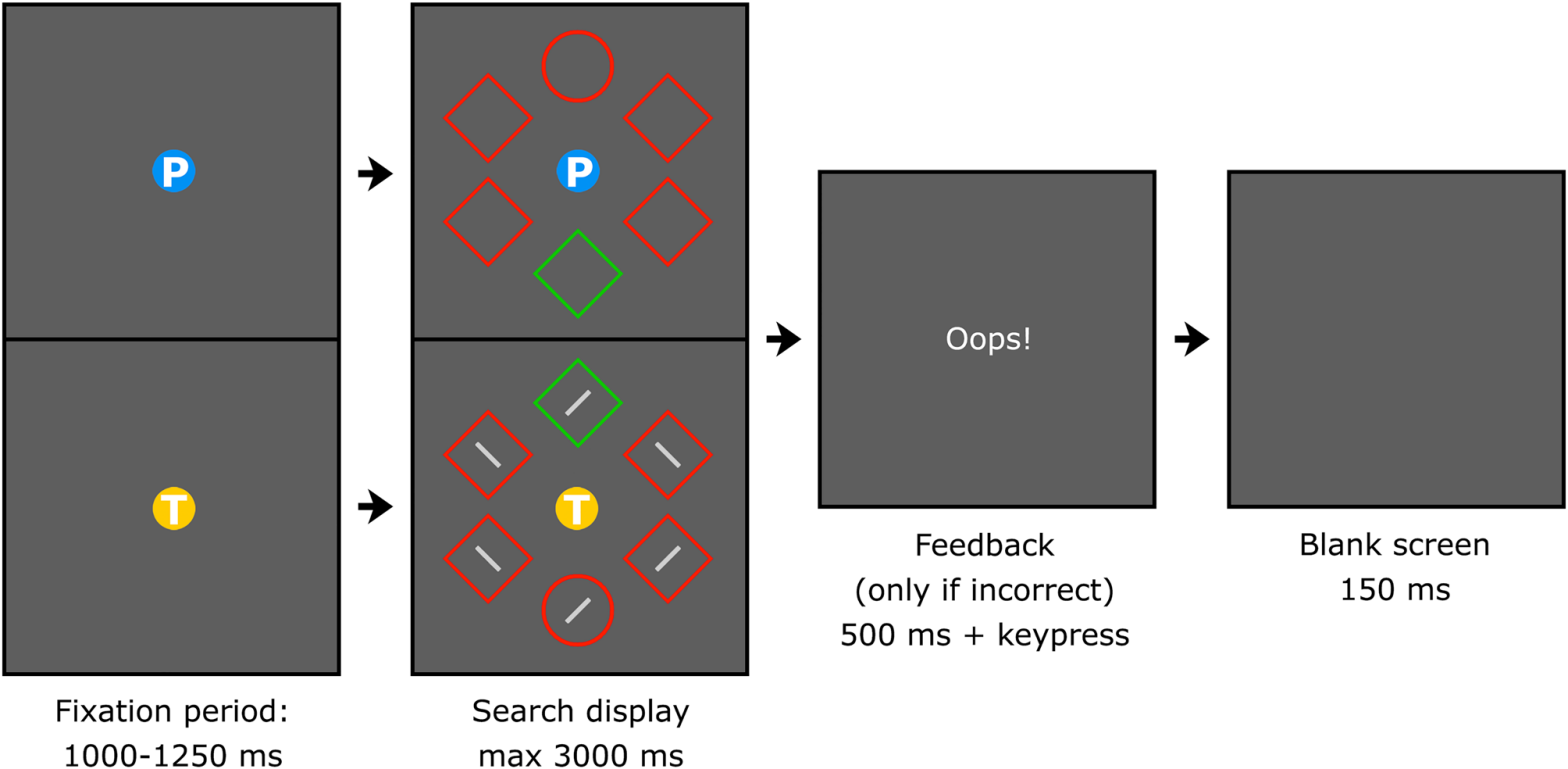
Schematic overview of a trial, with the top presenting the detection task and the bottom the compound search task. The search display was visible until a keyboard response was provided or the 3000 ms limit was exceeded. In the detection task participants reported whether a unique shape was present (left arrow key) or absent (right arrow key). In the compound search task participants indicated the tilt direction (left/right) of the line segment inside the unique shape. Incorrect or too slow responses were followed by the word ‘Oops!’ and a written instruction of the task. Figure created in Adobe Photoshop 22.4.3, https://www.adobe.com/products/photoshop.html.

The experimental display is illustrated in Fig. 2. Six shapes (one circle and five diamonds, or vice versa) were presented on an imaginary circle with a radius of 180 px. The circles and diamonds were 126 and 157 px high, respectively, in red (255/0/0) or green (0/200/0). Depending on the task, the center of the display contained a blue (37/146/242, radius: 14 px) or yellow (253/203/41) circle with a white P (for *presence*) or T (for *tilt,* Verdana Bold, height: 19 px) inside it, respectively. In the T-task (compound search), each shape contained a grey (128/128/128) 45° or − 45° line (57 × 8 px). The background was dark grey (94/94/94).

#### Procedure and design

Figure 2 gives a schematic overview of a trial. The duration of the fixation period was randomly selected between 1000 and 1250 ms. The search display was visible until response or until a 3000 ms limit was exceeded. Participants searched for a unique shape (i.e., a circle among diamonds or vice versa). In the detection task (cued by a blue P) they reported whether it was present (left arrow key) or absent (right arrow key). In the compound search task (cued by a yellow T) they indicated the tilt direction of the line segment embedded within that shape (left/right arrow key). The P or T was already visible during the fixation period in order to cue the task. If the response was incorrect or too slow, a brief error message (‘Oops!’) was shown for 500 ms, followed by a written instruction of the task until a keypress.

The target was always the uniquely shaped item, while the distractor was the uniquely colored item. A uniquely colored distractor was present on 50% of the trials. Whereas the target location was selected at random across all blocks, in the training blocks one distractor location occurred more often (72%) than the other locations (5.6% per location). The two high-probability locations were determined randomly for each participant, with the high-probability location of one task always opposite to that of the other task. The high-probability location for each task remained constant throughout the experiment. Critically, in testing blocks this spatial imbalance was removed and the distractor appeared with equal probability across all locations. In the compound search task, each shape in the display contained a line that was left or right-tilted at random. Participants were instructed to report the orientation of the line inside the target (i.e., the unique shape). In the detection task, the target was absent in half of the distractor-present trials and in half of the distractor-absent trials and participants were instructed to indicate whether or not the display contained a unique shape.

Figure 1A provides an overview of the block order. The experiment was separated into training blocks (72 trials) and testing blocks (144 trials). Participants performed a training block of task A, followed by a training block of task B and a testing block of task A. Next, they performed a training block of task B, followed by a training block of task A and testing block of task B. Before every block, participants were told if it was going to be a P-block (detection task) or a T-block (compound search task), so that a context shift could be anticipated prior to starting the first trial of a new block. Every training block started with a practice block of 20 trials, that was repeated until accuracy reached at least 70% (on average, participants performed each practice phase 1.06 times). Practice trials were included also in the second half of the experiment so that the total number of trials in which the regularity could be learned (this includes practice trials) in the first and second half of the experiment was approximately equal. A break was included after every block, and halfway through every testing block. The specific order of tasks was counterbalanced across participants so that half started with the compound search task (yellow T) and half started with the detection task (blue P). Awareness of the spatial regularities was assessed after all trials were completed. First, participants were asked whether the distractor appeared more frequently in one location than in other locations. Next, they were asked to indicate which location they thought was the high-probability location by typing in a location-based number (0–5), separately for each specific task (so twice in total, with a different central cue to indicate the task context).

#### Analyses

For response time (RT) analyses, we removed incorrect trials (5.2%) and performed a cleaning procedure (1.7%): RTs that deviated more than 3 SD from the mean (computed separately for each participant and each task) were also removed. Overall, this data cleaning procedure resulted in a loss of 6.9% of the data. The data for one participant was discarded because the overall accuracy was below 75%. As there was no evidence in support of a speed-accuracy trade-off (neither here, nor in the control experiment), we only report RT results in the main text. Accuracy results are reported in the supplementary analyses. For simplicity, we only report distractor-based analyses in the main text, but target-based analyses are reported in the supplementary analyses. Since half of the trials in the main experiment are not suitable for a target-based analysis^47^, we only performed those analyses on the control experiment. All *t*-tests are planned comparisons, unless stated otherwise^52^. If an analysis averaged across two tasks, an average was first calculated separately for each task, and then combined into a single average per participant. If no average could be computed for one of the two tasks, the data of that participant (one participant in Blocks 1 and 4) was omitted from the analysis. ANOVAs, *t*-tests, and Bayesian equivalents were performed using Jamovi^53^. For Bayesian analyses we used the default Cauchy distribution (scale = 0.707) as the prior. We report BF_10_ (expressing the strength of evidence in favor of the alternative hypothesis), meaning that BF_10_ < 1 is in favor of the null-hypothesis (with the strength of evidence increasing as the BF approaches zero). The verbal labels used to describe the strength of the evidence (in either direction) are based on an established classification^54,55^. A Greenhouse–Geisser correction was applied to the ANOVA results when the sphericity assumption was violated.

### Results

The main analyses are based on data that is collapsed across the two tasks to increase statistical power and focus on context-dependent location learning rather than the specifics of each task. Separate analyses for each task in the testing blocks can be found in the supplementary analyses (Fig. S2).

#### Training blocks

**Figure 3:**
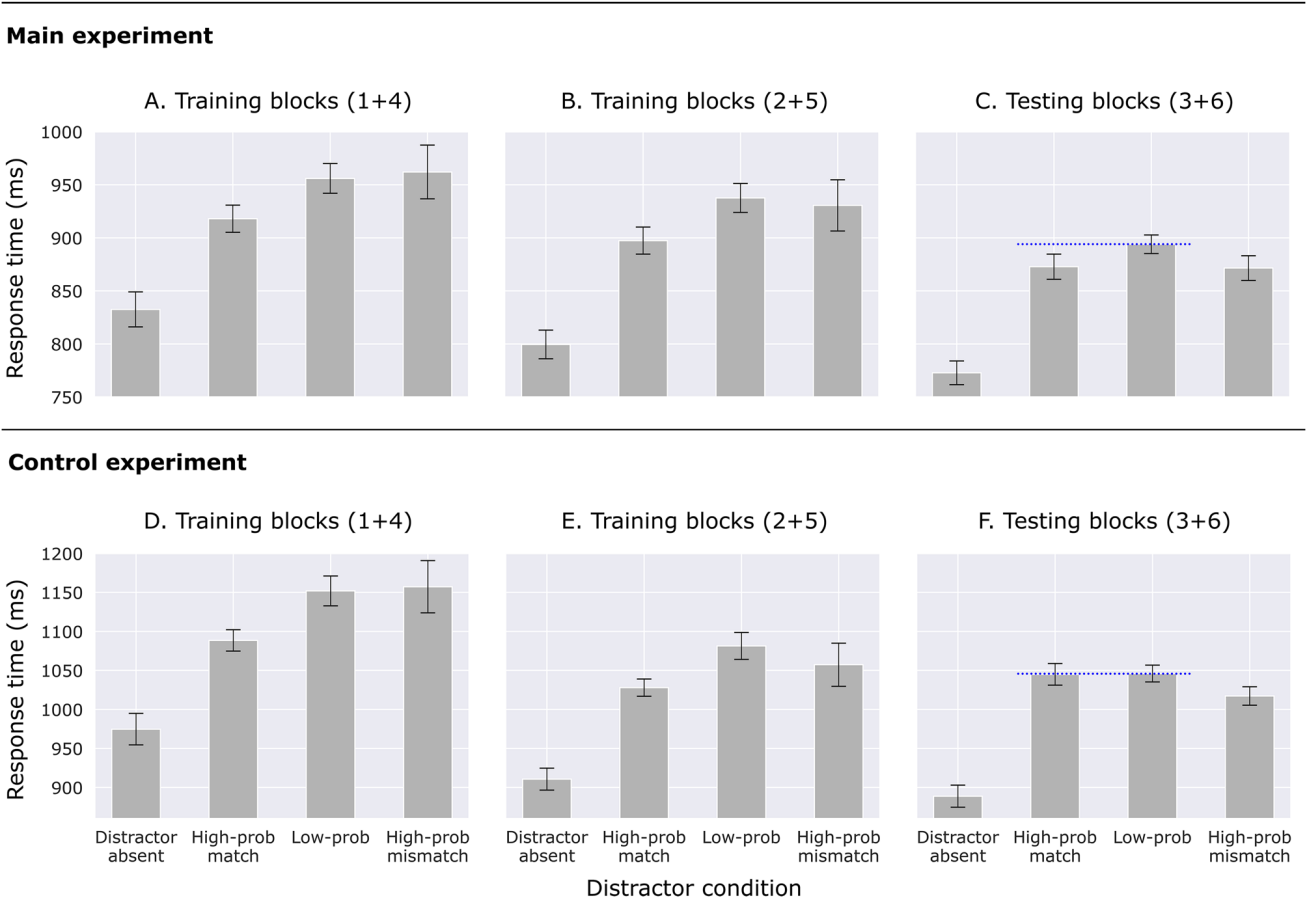
Response times as a function of distractor condition, separated by training and testing blocks, for both the main experiment (**A**–**C**) and the control experiment (**D**–**F**). Error bars indicate 95% within-subject confidence intervals^56^. (**A**,**D**) Training blocks: the first and fourth blocks. (**B**,**E**) Training blocks: the second and fifth blocks. (**C**,**F**) Testing blocks: the third and sixth blocks. The dotted blue line indicates the crucial difference between the main experiment and the control experiment.

Figure 3A and B show response times from the training blocks. The practice trials are also included in this analysis, because lingering suppression effects are expected to be strongest immediately following a switch (but including the practice trials does not change the pattern of results). The training blocks are split into Blocks 1 and 4, the first training blocks of each experiment half, and Blocks 2 and 5, the last training blocks before the testing blocks. The match condition refers to the high-probability location that is associated with the current task context, the mismatch condition refers to the high-probability location this is associated with the other task context, and the low-probability condition refers to the four remaining locations. It should be noted that the mismatch condition is based on only 4 trials (excluding practice trials) per participant, and therefore does not provide an accurate RT estimate.

In Blocks 1 and 4 (Fig. 3A), RTs differed significantly between distractor conditions (absent, match, low-probability, mismatch), *F*(1.85, 96.4) = 32.3, *p* < 0.001, *η*^*2*^_*p*_ = 0.38. Participants learned to suppress the current high-probability location, as responses were faster at the match location than the low-probability locations, *t*(52) = 3.55, *p* < 0.001, *BF*_*10*_ = 32.6, *d* = 0.49. The mismatch location was numerically the least suppressed location, but it did not differ significantly from the low-probability locations, *t*(52) = 0.36, *p* = 0.719, *BF*_*10*_ = 0.16, *d* = 0.49 (where the BF provides moderate evidence for the absence of a difference). It did differ from the match location, *t*(52) = 2.5, *p* = 0.016, *BF*_*10*_ = 2.49, *d* = 0.34 (although the BF is inconclusive).

In Blocks 2 and 5 (Fig. 3B), RTs also differed significantly between distractor conditions, *F*(1.87, 98.95) = 41.9, *p* < 0.001, *η*^*2*^_*p*_ = 0.44. Participants learned to suppress the current high-probability location, as responses were faster at the match location than the low-probability locations, *t*(53) = 4.14, *p* < 0.001, *BF*_*10*_ = 181, *d* = 0.56. Numerically, the mismatch location potentially shows some lingering suppression from the previous block, but it was not significantly different from the low-probability locations, *t*(53) = 0.42, *p* = 0.679, *BF*_*10*_ = 0.16, *d* = 0.06 (where the BF provides moderate evidence for the absence of a difference). It approaches significance in comparison to the match location, *t*(53) = 1.92, *p* = 0.06, *BF*_*10*_ = 0.82, *d* = 0.26 (where the BF is inconclusive).

#### Testing blocks

Figure 3C shows response times from the testing blocks. RTs differed significantly between distractor conditions, *F*(2.56, 135.52) = 70.9, *p* < 0.001, *η*^*2*^_*p*_ = 0.57. Comparison to the low-probability locations shows that participants suppressed both the match location, *t*(53) = 2.93, *p* = 0.005, *BF*_*10*_ = 6.62, *d* = 0.4, and the mismatch location, *t*(53) = 2.51, *p* = 0.015, *BF*_*10*_ = 2.57, *d* = 0.34 (although the BF was inconclusive). There was no difference between the match and mismatch locations, *t*(53) = 0.12, *p* = 0.907, *BF*_*10*_ = 0.15, *d* = 0.02. Separate analyses for each task are shown in Fig. S2 in the supplementary analyses.

#### Revival of suppression

**Figure 4:**
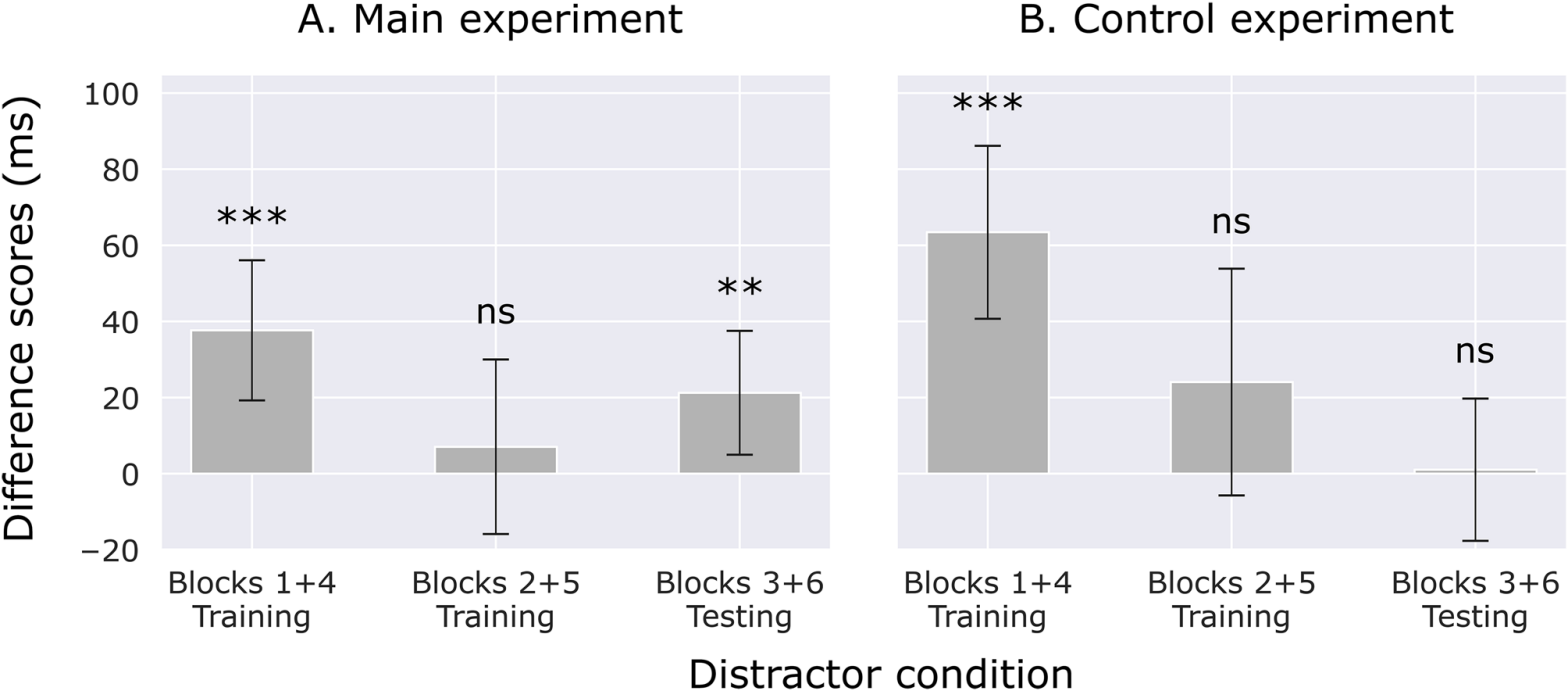
Difference scores between the first-learned location (i.e., location A in the first half of the experiment, and location B in the second half) and the low-probability locations, as a function of block type, for the main experiment (**A**) and the control experiment (**B**). Error bars indicate 95% within-subject confidence intervals^56^.

A crucial finding in the response time analyses is that the suppression for the first-learned location (i.e., location A in the first half of the experiment, and location B in the second half) disappears when the task context changes and a second high-probability location is introduced (in the second and fifth blocks), but reappears when the first-learned context is encountered again in the testing block. To focus on this revival of suppression, Fig. 4A shows the difference scores between the first-learned location and the low-probability locations across blocks. We argue that this revival of suppression is driven by the retrieval of the first-learned location from memory, based on its associated context (see also Fig. 1B). This also means that learning took place in a context-dependent way.

#### Awareness of the regularities

One-third of the participants (33%, here called the ‘aware’ group) indicated that the distractor occurred more often at some locations than others. Participants were also asked to indicate the high-probability location for each of the two tasks. By taking the distance (ranging from 0 to 3) between the location indicated by the participant and the actual high-probability location for each of the two tasks, and averaging across the two distances, an awareness score was computed for every participant. An awareness score below chance-level could indicate some level of awareness of the high-probability locations.

We first computed a ‘context-blind’ awareness score, only looking at whether participants could indicate the two high-probability locations, independent of context. In this computation, if a participant selected location A for context B and location B for context A, we regarded that as a perfect score (0). This context-blind awareness score (mean = 0.94, SD = 0.52) was not below chance-level (0.87), *t*(53) = 0.82, *p* = 0.818, *BF*_*10*_ = 0.08, *d* = 0.13 (the BF in fact provides strong evidence for chance-level performance). Furthermore, the score also did not differ significantly from chance for the ‘aware’ group, *t*(17) = 1.07, *p* = 0.851, *BF*_*10*_ = 0.130, *d* = 0.25, or the ‘unaware’ group, *t*(35) = 0.37, *p* = 0.643, *BF*_*10*_ = 0.14, *d* = 0.06. Lastly, the ‘aware’ and ‘unaware’ groups did not differ in terms of their awareness score, *t*(52) = 0.6, *p* = 0.525, *BF*_*10*_ = 0.34, *d* = 0.19.

We also computed a ‘context-dependent’ awareness score, looking at whether participants could indicate the correct high-probability location for each of the two contexts separately. In this computation, if a participant selected location A for context B and location B for context A, we regarded that as the worst possible score (3), because each selected location was 3 positions away from the context-dependent location. This context-dependent awareness score across participants (mean = 1.6, SD = 0.77) was not below chance-level (1.5), *t*(53) = 0.98, *p* = 0.833, *BF*_*10*_ = 0.08, *d* = 0.08 (the BF in fact provides strong evidence for chance-level performance). Furthermore, the awareness score also did not differ significantly from chance for the ‘aware’ group, *t*(17) = 1.76, *p* = 0.952, *BF*_*10*_ = 0.101, *d* = 0.42, or the ‘unaware’ group, *t*(35) = 0.54, *p* = 0.541, *BF*_*10*_ = 0.17, *d* = 0.02. Lastly, the ‘aware’ and ‘unaware’ groups did not differ in terms of their awareness score, *t*(52) = 1.2, *p* = 0.237, *BF*_*10*_ = 0.51, *d* = 0.35. We conclude that there was likely no or very little awareness of the learned regularities, although the used measure of awareness should be interpreted with caution^57–59^.

### Discussion

The results of the main experiment were not in line with our predictions for context-dependent learning, nor our predictions for context-independent learning. On the one hand, there appears to be some context-dependent learning, as the high-probability location that was learned in Blocks 1 and 4 was no longer suppressed in Blocks 2 and 5, while suppression for this location (match) resurfaced in the testing blocks. Since all distractor locations were equiprobable in the testing blocks, this resurfaced suppression appears to be the result of context-dependent learning: upon recognizing a previously learned task context, the associated high-probability location was suppressed (see also Fig. 4A). However, we also observed lingering suppression for the mismatch location in the testing blocks, and this was not in line with our prediction of context-dependent learning.

## Control experiment

To be able to better understand the revival of suppression in the testing blocks and the role of context therein, we ran a control experiment that followed the same experimental design, except that we removed all context switches. Participants performed the same task (the compound search task) throughout the experiment. Crucially, the high-probability distractor location changed between blocks in exactly the same way as in the main experiment. That is, the order of high-probability locations across blocks was: A, B, none (testing block), B, A, none (testing block). To facilitate comparison between the main experiment and the control experiment, we labelled the conditions the same way as in the main experiment, as if the high-probability locations were matching or mismatching the context (see Fig. 1A). We reasoned that if we again observed revival in this control experiment, we could rule out context-dependent learning. If we found a different pattern of results, that difference could be attributed to the switches in context and would indicate context-dependent learning in the main experiment. In the main experiment, a separate analysis of the compound search task yielded significant suppression for the match location (Fig. S2A), such that the difference between the main experiment and the control experiment cannot be ascribed to the characteristics of the detection task.

### Methods

Fifty-five adults (30 male, 23 female, 2 non-binary, mean age = 32, age range: 21 to 50) participated. The procedure was identical to that of the main experiment, with the exception that the task remained the same throughout the experiment (i.e., a compound search task). As in the main experiment, practice blocks were repeated until accuracy reached at least 70% (on average, participants performed each practice phase 1.08 times).

#### Analyses

The analyses were identical to the main experiment with the exception that we did not separate the analyses per context. While there were no actual context changes in the control experiment, we use the same condition labels as in the main experiment. Thus, ‘match’ refers to the location that would have been matching the context if there was one. Furthermore, the control experiment also allowed for analyzing response times in relation to the target location, because the compound search task lends itself to that analysis. Those results are reported in the supplementary analyses. Removal of incorrect trials (4.4%) and RT filtering (1.4%) resulted in an overall loss of 5.8% of the data.

### Results

#### Training blocks

Figure 3D and E show response times from the training blocks. The results from the training blocks for the control experiment follow the pattern of results of the main experiment. In Blocks 1 and 4, RTs differed significantly between distractor conditions, *F*(1.75, 94.55) = 39.9, *p* < 0.001, *η*^*2*^_*p*_ = 0.43. Participants learned to suppress the current high-probability location, as responses were faster at the match location than the low-probability locations, *t*(54) = 5.61, *p* < 0.001, *BF*_*10*_ = 21,873, *d* = 0.76. The mismatch location was numerically the least suppressed location, but it did not differ significantly from the low-probability locations, *t*(54) = 0.22, *p* = 0.826, *BF*_*10*_ = 0.15, *d* = 0.03 (where the BF provides moderate evidence for the absence of a difference). It did differ from the match location, *t*(54) = 3.05, *p* = 0.004, *BF*_*10*_ = 8.94, *d* = 0.41.

In Blocks 2 and 5, RTs also differed significantly between distractor conditions, *F*(1.68, 90.66) = 47.8, *p* < 0.001, *η*^*2*^_*p*_ = 0.47. Participants learned to suppress the current high-probability location, as responses were faster at the match location than the low-probability locations, *t*(54) = 5.54, *p* < 0.001, *BF*_*10*_ = 17,205, *d* = 0.75. Numerically, the mismatch location potentially shows some lingering suppression from the previous block, but it was not significantly different from the low-probability locations, *t*(54) = 1.15, *p* = 0.256, *BF*_*10*_ = 0.27, *d* = 0.15 (where the BF provides moderate evidence for the absence of a difference). The comparison between match and mismatch was also nonsignificant, *t*(54) = 1.59, *p* = 0.118, *BF*_*10*_ = 0.48, *d* = 0.21.

#### Testing blocks

Figure 3F shows response times from the testing blocks. RTs differed significantly between distractor conditions, *F*(3, 162) = 99.5, *p* < 0.001, *η*^*2*^_*p*_ = 0.65. In contrast with the main experiment, participants did not suppress the match location compared to the low-probability locations, *t*(54) = 0.11, *p* = 0.916, *BF*_*10*_ = 0.15, *d* = 0.01 (where the BF provides moderate evidence for the absence of a difference). In line with the main experiment, they did suppress the mismatch location, *t*(54) = 3.27, *p* = 0.002, *BF*_*10*_ = 15.61, *d* = 0.44. The difference between the match and mismatch locations was significant, *t*(54) = 2.48, *p* = 0.016, *BF*_*10*_ = 2.36, *d* = 0.33 (although the BF was inconclusive).

#### Awareness of the regularities

As in the main experiment, approximately one-third of the participants (36%, here called the ‘aware’ group) indicated that the distractor occurred more often at some locations than others. Since context in the control experiment was not differentiated, we only computed a ‘context-blind’ awareness score. In contrast to the main experiment, this context-blind awareness score (mean = 0.74, SD = 0.5) was below chance-level (0.87), *t*(54) = 1.99, *p* = 0.026, *BF*_*10*_ = 1.77, *d* = 0.27 (though the BF was inconclusive). However, the score was not significantly below chance for the ‘aware’ group, *t*(19) = 1.60, *p* = 0.063, *BF*_*10*_ = 1.28, *d* = 0.36, or the ‘unaware’ group, *t*(34) = 1.23, *p* = 0.114, *BF*_*10*_ = 0.64, *d* = 0.21. The ‘aware’ and ‘unaware’ groups did not differ in terms of their awareness score, *t*(53) = 0.69, *p* = 0.192, *BF*_*10*_ = 0.34, *d* = 0.19.

### Discussion

The response time results of the control experiment were in line with the main experiment, except for one crucial difference: there was no revival of suppression at the matching location in the testing block (see also Fig. 4B). We interpret this finding as follows. Given the alternating task context across blocks (see Fig. 1A), the match location in the testing blocks was learned first (in the first and fourth blocks), while the mismatch location in the testing blocks was learned second (in the second and fifth blocks), right before the testing blocks. The suppression for the match location was overridden by the new high-probability location in the second and fifth blocks, and the suppression of this location continued in the testing blocks. Crucially, the suppression at the match location was not revived in the testing blocks, because there was no context to actually enforce such a revival. Note that when the main experiment and the control experiment are compared on the basis of the compound search task alone, the same interpretation holds, because separate analysis of the compound search task yielded significant suppression at the context-matching location (Fig. S2A), similar to the collapsed analysis (Fig. 3C).

In contrast to the main experiment, the control experiment provided some evidence of awareness of the high-probability distractor locations. It should be noted however that the awareness score is based on only two responses, and therefore may not provide a reliable estimate. Previous studies^57–59^ have argued that common post-experiment awareness questionnaires are insufficient to conclude unawareness, but this argument also works in the other direction. The measure is simply so unreliable that it is questionable whether it is meaningful at all. Perhaps unsurprisingly then, the Bayesian analyses were inconclusive, and the awareness score was no longer significant when we only included participants that actually indicated an imbalance in the distractor distribution (the ‘aware’ group) when asked about this. We conclude that the difference between the main experiment and the control experiment is unlikely to be due to a difference in awareness of the distractor regularities.

## General discussion

Statistical regularities regarding the location of distractors in visual displays have been shown to facilitate search. This is because humans can learn to suppress locations that are more likely to contain a distractor^11,13,14,47^. Somewhat surprisingly, a series of recent findings suggested that this form of learned suppression is insensitive to context^44,45^. The current study however presents a different picture: we show that participants can actually learn to suppress a location in a context-dependent way, at least so long as each context is presented for an extended period of time (i.e., blocks of trials), and the contexts are dissimilar enough. We were able to uncover these findings by using a blocked design with separate training and testing blocks (Fig. 1A), in contrast to previous studies which used a mixed design^44,45^. Furthermore, as a means of differentiating contexts more strongly than previous studies, the task alternated from block to block between a compound search task and a detection task. Crucially, a different high-probability distractor location was assigned to each context in the training blocks, and all distractor locations were made equiprobable in the testing blocks. In the control experiment, the contexts were made indistinguishable for participants, but the high-probability locations changed in exactly the same way as in the main experiment. Upon encountering a familiar task context in a testing block, participants showed a revival of suppression for the location that was associated with that context in an earlier training block. Crucially, this revival of suppression for a familiar context was absent in the control experiment, simply because there were no contexts to associate the distractor locations with.

Our findings are the first evidence for context-dependent spatial suppression resulting from statistical learning, providing a novel and important insight into the mechanisms behind the implicit learning of distractor locations. The finding that distractor location probabilities can be learned in a context-dependent way is crucial for the relevance of history-based attentional effects. Without some form of contextual binding, learned suppression effects would require a constant re-learning of the statistical regularities of contexts that have already been encountered. On a more theoretical level, context-dependency can be taken as evidence for an intelligent learning mechanism that maintains multiple regularities over time, as opposed to a simple habit-like mechanism where only the last-learned regularity is available. Furthermore, our findings reconcile the previously observed generalized suppression^44,45^ with the presumed context-dependency of selection history effects^5^ and the observed context-dependency in the reward learning^31^, contextual cueing^35^, and broader statistical learning literature^41,42^. However, our findings do not imply that the learning of multiple distractor regularities is always context-dependent. It remains to be seen whether weaker context manipulations such as background images can also instantiate context-dependent effects when applied in a blocked design. Context switches may simply need to be sufficiently spaced in time for context-dependent learning to occur, because otherwise two contexts would need to be updated more or less in parallel. It is also possible that in mixed designs^44,45^ context-dependent learning did occur but was obscured in the data by lingering suppression effects.

Besides context-dependent learning we also observed lingering suppression from the directly preceding context. As described, testing blocks yielded a revival of earlier suppression matching the context of the testing block, but there was also suppression that mismatched with the testing block’s context (although the BF was inconclusive in the main experiment). In other words, both the high-probability distractor locations were suppressed in the testing blocks. This was a puzzling finding, because if learning was context-dependent, we expected that suppression would only be active for the location matching the current context. If a location-context association has been learned, it seems a waste of energy to suppress more than what this association dictates. We interpret the mismatching suppression in the testing blocks as lingering suppression from the directly preceding training block. This raises another question: Why would the suppression from the previous training block linger in the testing blocks, but not (or much weaker) in the training blocks? The simplest answer appears to be that a different high-probability location is introduced in the training blocks, whereas the testing blocks contain no high-probability locations whatsoever. Thus, we explain the data as follows: Participants can implicitly learn to associate high-probability distractor locations with their respective contexts, but suppression for a previous high-probability location continues to linger unless a new high-probability location is introduced^13^. It seems unlikely that suppression will linger indefinitely, so we assume that it gradually decreases, but the 144 trials in our testing blocks were not enough to show this.

Lingering suppression could result from a slow neural mechanism that requires time to adapt to a new situation. The neural underpinnings of implicitly learned suppression are still poorly understood^27,60–63^ so that many options remain open. In addition to or instead of neural factors, lingering suppression likely reflects the inherent slowness of statistical learning, where repetitions are crucial to filter out the noise in an environment that is riddled with randomness. However, neither of these explanations appear entirely satisfactory in this case, because they cannot explain the stark difference between the strong presence of lingering suppression in the testing blocks, and the absence or near absence of lingering suppression in the training blocks. The low trial counts in crucial conditions unfortunately make it impossible to investigate the time course of learning within blocks, but the averaged results per block suggest that the 72 trials + 20 practice trials of the training blocks were enough to remove most or all lingering suppression, whereas the 144 trials of the testing blocks did very little (if anything) to decrease it. Thus, it appears that the priority map can be quickly updated when a new high-probability location is introduced^64^, but updates slowly when all spatial imbalances are removed^44,65,66^. Part of the answer might lie in the trial counts at the mismatch location specifically (where lingering suppression shows up), because those are only 2 trials per training block and 12 per testing block. The low trial count at the mismatch location in the training blocks could have made it difficult to let go of previous suppression, or it could simply mean that the response time for that specific condition was unreliable. In general, findings on the time course of learning and unlearning often do not converge, suggesting that many factors play a role^11,44,66–69^.

In sum, the present findings show that statistical learning of spatial suppression is context dependent, at least so long as the contexts are dissimilar enough and presented in blocks. However, we also observed lingering suppression for locations mismatching with the context. A control experiment with no contextual differentiation whatsoever confirmed that learning in the main experiment must have been context-dependent. The long-lasting lingering suppression is a finding that requires further explanation, and we have made several suggestions for avenues of further investigation. Our findings substantially increase the real-life relevance of implicitly learned attentional suppression. Naturally, the present results are limited in that they only consider distractor suppression as opposed to target enhancement, and implicit as opposed to explicit learning. Furthermore, we have operationalized context by using two similar but distinct tasks, and it remains to be seen whether context-manipulations that are not task-relevant can also be learned in a context-dependent way^70,71^, and whether context-manipulations that are even stronger might result in less lingering suppression. In this sense, the current study raises a series of interesting questions about the learning and lingering of context-dependent regularities in visual search as well as in other domains of cognition, which could be explored in future research.

## Supplementary Information

Supplementary Information.

## Data Availability

Experimental materials and data are available here: https://osf.io/xm59c/. None of the experiments were preregistered.
