# Supplementary material for: Statistical learning of distractor locations is dependent on task context
Source: Sci Rep. 2023 Jul 11;13:11234. doi: 10.1038/s41598-023-38261-z (PMC10336038; doi:10.1038/s41598-023-38261-z)
Supplement: Supplementary file 1 — Supplementary Information. [file 41598_2023_38261_MOESM1_ESM.docx]

**Statistical learning of distractor locations is dependent on task context**

Jasper de Waard*^1,2^, Dirk van Moorselaar^1,2^, Louisa Bogaerts^1,2,3^, Jan Theeuwes^1,2,4^

^1^Department ­­of Experimental and Applied Psychology,

Vrije Universiteit Amsterdam, Amsterdam, the Netherlands

^2^Institute Brain and Behavior Amsterdam (iBBA)

^3^Department of Experimental Psychology, Ghent University, Ghent, Belgium

^4^William James Center for Research, ISPA-Instituto Universitario, Lisbon, Portugal

**Supplementary analyses**

*Error rates*

Figure S1 shows the error rates for the main experiment and the control experiment. Error rates could point towards a speed-accuracy trade-off if the pattern of results goes in a different direction than the response time results (Figure 3). The pattern of results is largely consistent with the response times and all inconsistencies, discussed in the remainder of this paragraph, were nonsignificant. In Figure S1A and D, there was a decrease in errors for the mismatch location compared to the low-probability locations which was nonsignificant for the main experiment, *t*(53) = 0.57, *p = .*573, *BF_10_* = 0.17, *d* = 0.08, and for the control experiment, *t*(54) = 0.64, *p = .*527, *BF_10_* = 0.18, *d* = 0.09. Figure S1E shows a decrease in errors for the mismatch location compared to the low-probability locations which was nonsignificant, *t*(54) = 0.47, *p = .*644, *BF_10_* = 0.16, *d* = 0.06. In all these cases, the difference was not only nonsignificant, but the BF in fact provided moderate evidence for the absence of a difference. Figure S1F shows a decrease in errors for the match location compared to the low-probability locations which was nonsignificant, *t*(54) = 1.58, *p = .*12, *BF_10_* = 0.47, *d* = 0.21, where the BF was inconclusive. Overall, we conclude that the response time results in the main experiment and in the control experiment cannot be ascribed to speed-accuracy trade-offs.


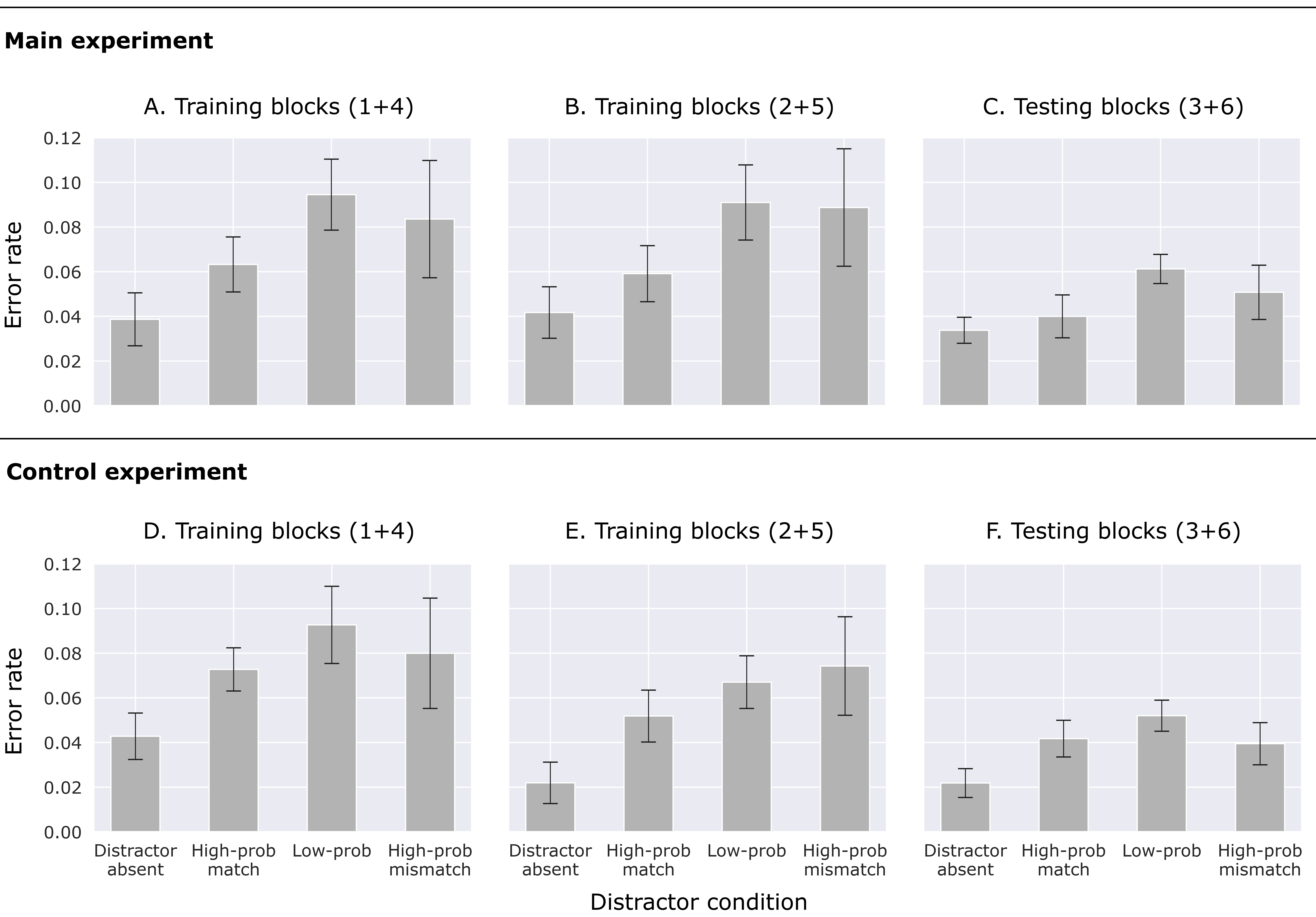


Figure S1: Error rates as a function of distractor condition, separated by training and testing blocks, for both the main experiment (ABC) and the control experiment (DEF). Error bars i­ndicate 95% within-subject confidence intervals ^1^. A&D) Training blocks: the first and fourth blocks. B&E) Training blocks: the second and fifth blocks. C&F) Testing blocks: the third and sixth blocks.

*Separate tasks*

The response times for the main experiment are averaged across two tasks: a compound search task and a visual detection task. This makes the analyses easier to comprehend and ensures that the conditions in the testing blocks are made up of enough trials (twenty-four each for the match and mismatch conditions). Figure S2 shows that both tasks share a very similar pattern of results. However, by splitting the analysis between the tasks, the trial counts are halved (twelve each for the match and mismatch conditions), and the resulting data lacks statistical power. In the compound search task (Figure S2A), comparison to the low-probability locations shows that participants suppressed both the match location, *t*(53) = 2.3, *p = .*025, *BF_10_* = 1.67, *d* = 0.31, and the mismatch location, *t*(53) = 2.01, *p = .*05, *BF_10_* = 0.95, *d* = 0.27 (although both BFs were inconclusive). In the detection task (Figure S2B), comparison to the low-probability locations was nonsignificant for the match location, *t*(53) = 1.88, *p = .*065, *BF_10_* = 0.77, *d* = 0.26, and the mismatch location, *t*(53) = 1.67, *p = .*1, *BF_10_* = 0.55, *d* = 0.23.


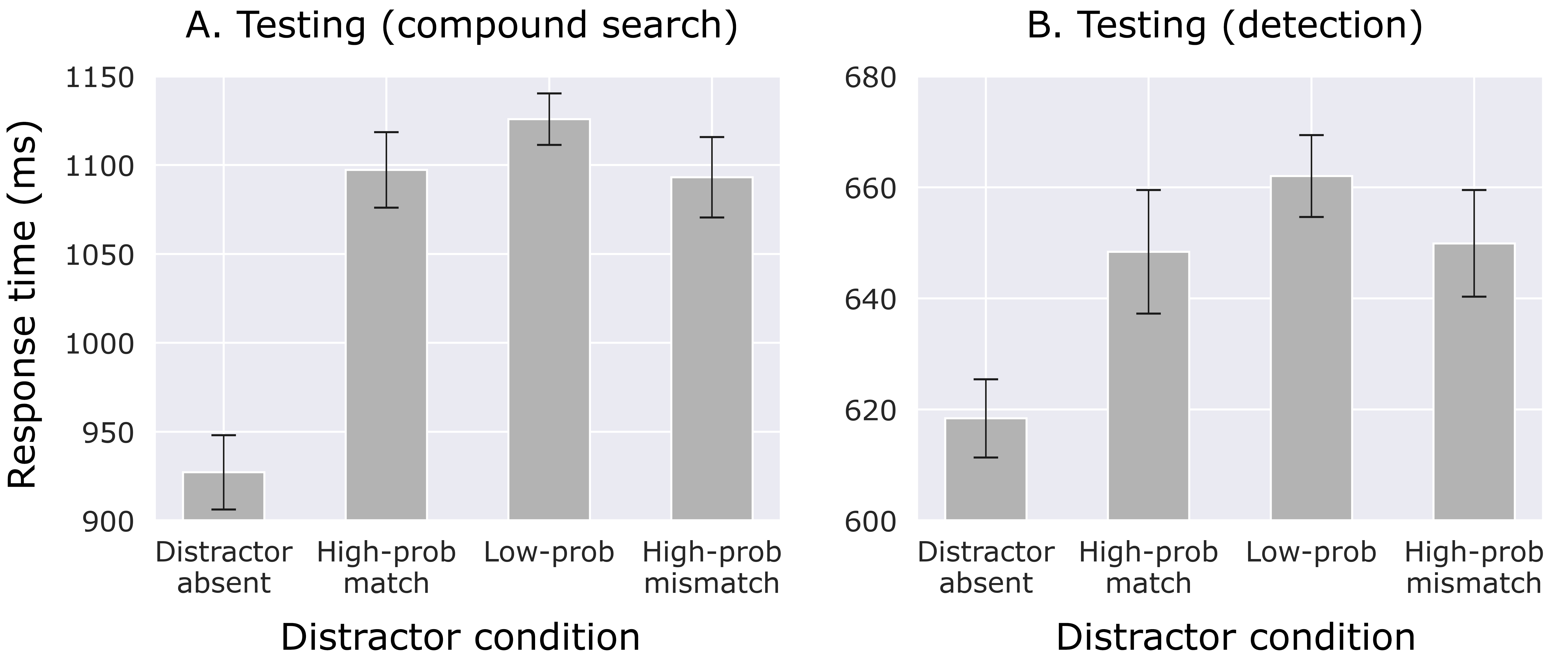


Figure S2: Response times as a function of distractor condition in the testing blocks of the main experiment, separately for the compound search (A) and detection (B) tasks. Error bars indicate 95% within-subject confidence intervals ^1^.

*Target-based suppression effects*

The effects of statistically learned distractor suppression in the additional singleton paradigm can also be observed in distractor-absent trials, such that responses are slowed when the target appears at the high-probability distractor location ^2^. However, those effects have not been observed in detection tasks ^3^, which made up half of the main experiment. For this reason, we analyze the target-based effects (Figure S3) only for the control experiment.


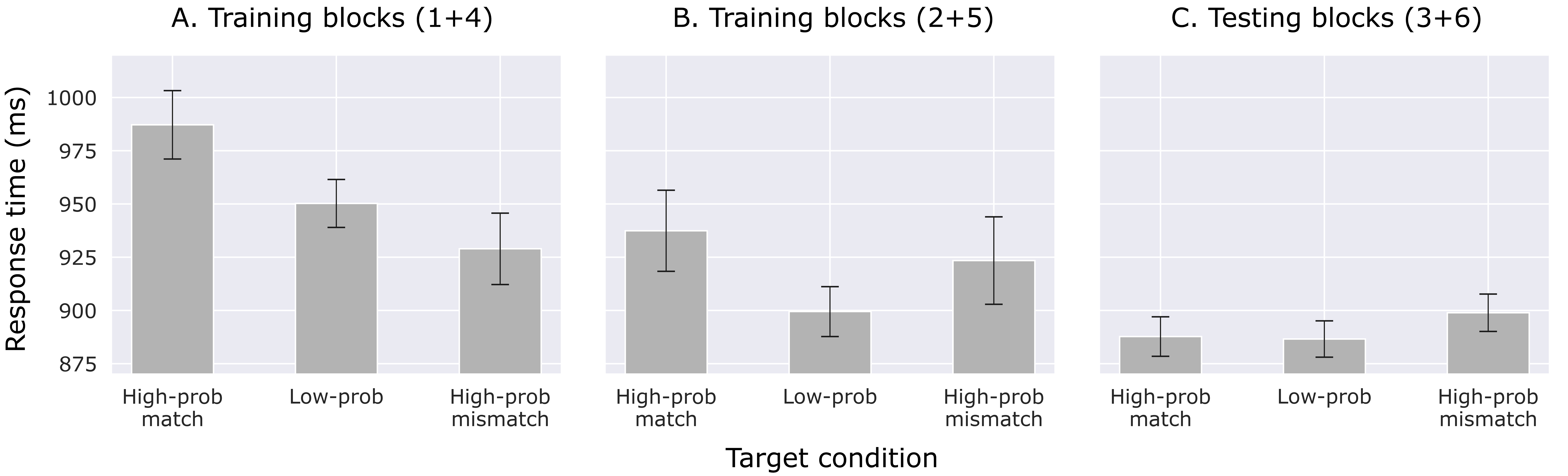


Figure S3: Response times as a function of target condition in distractor-absent trials of the control experiment. Error bars indicate 95% within-subject confidence intervals ^1^. A) Training blocks: the first and fourth blocks. B) Training blocks: the second and fifth blocks. C) Testing blocks: the third and sixth blocks.

Figure S3A and B show response times from the training blocks. In Blocks 1 and 4 (Figure S3A), RTs differed significantly between distractor conditions, *F*(1.68, 90.97) = 10, *p* < .001, *η^2^_p_* = 0.16. In line with the distractor-based results, responses were slower at the match location than the low-probability locations, *t*(54) = 3.28, *p < .*002, *BF_10_* = 16.2, *d* = 0.44. The mismatch location was numerically the least suppressed location, but it did not differ significantly from the low-probability locations, *t*(54) = 1.77, *p = .*083, *BF_10_* = 0.63, *d* = 0.24. It did differ from the match location, *t*(54) = 3.71, *p < .*001, *BF_10_* = 51.75, *d* = 0.5.

In Blocks 2 and 5 (Figure S3B), RTs also differed significantly between distractor conditions, *F*(1.33, 72) = 4.92, *p* = .02, *η^2^_p_* = 0.08. In line with the distractor-based results, responses were slower at the match location than the low-probability locations, *t*(54) = 3.09, *p = .*003, *BF_10_* = 9.98, *d* = 0.42. The mismatch location potentially shows some lingering suppression from the previous block, but it was not significantly different from the low-probability locations, *t*(54) = 1.71, *p = .*093, *BF_10_* = 0.57, *d* = 0.23. The comparison between match and mismatch is also nonsignificant, *t*(54) = 0.73, *p = .*471, *BF_10_* = 0.19, *d* = 0.1.

Figure S3C shows response times from the testing blocks. RTs did not differ significantly between target conditions, *F*(2, 108) = 1.51, *p* = .226, *η^2^_p_* = 0.03. This could suggest that the effects of distractor-based regularities on the target wane relatively quickly once those regularities have disappeared.

**References**

1. Cousineau, D. Confidence intervals in within-subject designs: A simpler solution to Loftus and Masson’s method. *Tutor. Quant. Methods Psychol.* **1**, 42–45 (2005).

2. Wang, B. & Theeuwes, J. Statistical regularities modulate attentional capture. *J. Exp. Psychol. Hum. Percept. Perform.* **44**, 13 (2018).

3. van Moorselaar, D. & Theeuwes, J. Spatial suppression due to statistical regularities in a visual detection task. (2021).
